# Utilizing “Omic” Technologies to Identify and Prioritize Novel Sources of Resistance to the Oomycete Pathogen *Phytophthora infestans* in Potato Germplasm Collections

**DOI:** 10.3389/fpls.2016.00672

**Published:** 2016-05-27

**Authors:** Pauline S. M. Van Weymers, Katie Baker, Xinwei Chen, Brian Harrower, David E. L. Cooke, Eleanor M. Gilroy, Paul R. J. Birch, Gaëtan J. A. Thilliez, Alison K. Lees, James S. Lynott, Miles R. Armstrong, Gaynor McKenzie, Glenn J. Bryan, Ingo Hein

**Affiliations:** ^1^Cell and Molecular Sciences, The James Hutton InstituteDundee, UK; ^2^Information and Computational Sciences, The James Hutton InstituteDundee, UK

**Keywords:** germplasm collection, Commonwealth potato collection, diagnostic, RenSeq, *Phytophthora infestans*, oomycete, RXLR effectors

## Abstract

The greatest threat to potato production world-wide is late blight, caused by the oomycete pathogen *Phytophthora infestans*. A screen of 126 wild diploid *Solanum* accessions from the Commonwealth Potato Collection (CPC) with *P. infestans* isolates belonging to the genotype 13-A2 identified resistances in the species *S. bulbocastanum, S. capsicibaccatum, S. microdontum, S. mochiquense, S. okadae, S. pinnatisectum, S. polyadenium, S. tarijense*, and *S. verrucosum.* Effector-omics, allele mining, and diagnostic RenSeq (dRenSeq) were utilized to investigate the nature of resistances in *S. okadae* accessions. dRenSeq in resistant *S. okadae* accessions 7129, 7625, 3762, and a bulk of 20 resistant progeny confirmed the presence of full-length *Rpi-vnt1.1* under stringent mapping conditions and corroborated allele mining results in the accessions 7129 and 7625 as well as Avr-vnt1 recognition in transient expression assays. In contrast, susceptible *S. okadae* accession 3761 and a bulk of 20 susceptible progeny lacked sequence homology in the 5′ end compared to the functional *Rpi-vnt1.1* gene. Further evaluation of *S. okadae* accessions with *P. infestans* isolates that have a broad spectrum of virulence demonstrated that, although *S. okadae* accessions 7129, 7625, and 7629 contain functional *Rpi-vnt1.1*, they also carry a novel resistance gene. We provide evidence that existing germplasm collections are important sources of novel resistances and that “omic” technologies such as dRenSeq-based genomics and effector-omics are efficacious tools to rapidly explore the diversity within these collections.

## Introduction

Potato is the most important non-cereal food crop worldwide and is consumed by more than a billion people (Birch et al., 2012). Global potato production between 1991 and 2007 has shown an increase of 21% that is driven by a 48% rise of potato production in the developing world, where the growing area has increased alongside yield. Pests and pathogens represent a serious and continuing threat to potato production, and the most widespread and economically significant of these is late blight, caused by the oomycete pathogen *Phytophthora infestans*. In agricultural systems major population changes of *P. infestans* lineages have been observed that often impact negatively on crop production. For example, in the European *P. infestans* population a new clonal lineage referred to as 13-A2 or “blue 13” was first detected in 2004 and, upon its arrival in Great Britain, came to dominate the population within 3 years (Cooke et al., 2012). Previously resistant potato cultivars such as Lady Balfour and Stirling were susceptible to the 13-A2 lineage and are consequently no longer suitable for the organically grown potato market. A conservative estimate of the chemical control costs and yield losses associated with late blight exceeds €6.7 Billion (Haverkort et al., 2009). In many parts of the world fungicide application is the only means to prevent disease. Predictions suggest that global potato production could exceed 400 Mt per year if diseases that reduce yields by ~25% could be controlled (Agrios, 1997).

The ability to withstand multiple biotic and abiotic stresses is critical for wild potato species, suggesting that many untapped, natural sources of resistance exist for exploitation in breeding programs. With the availability of extensive germplasm resources, including the Commonwealth Potato Collection (CPC) at the James Hutton Institute (Bradshaw et al., 2006), and improved genomics tools, the potential to exploit this natural biodiversity is considerable. Newly identified and deployed resistances could provide an environmentally benign opportunity to secure potatoes as a major food source in the future (Birch et al., 2012). Critical for the success of such disease control is, however, a detailed knowledge of the underlying mechanisms of defense to facilitate complementary deployment of resistances.

Inducible resistance responses in plants require the direct or indirect detection of pathogen molecules such as defense elicitors or effector molecules via plant receptors (Jones and Dangl, 2006; Wiesel et al., 2014). Effectors, once recognized, are known as avirulence (*Avr*) genes as their recognition often yields incompatibility for the pathogen on plants that carry the cognate resistance (R) protein. Genome-wide analysis of *P. infestans* and other oomycetes has shown that all identified *Avr* genes contain a canonical RXLR motif, which has led to coining of the term RXLR effectors (Armstrong et al., 2005; Hein et al., 2009; Raffaele et al., 2010; Cooke et al., 2012). Heterologous expression of these effectors is used as a novel tool for the identification of resistances and for disease resistance breeding (Birch et al., 2008; Vleeshouwers and Oliver, 2014; Lenman et al., 2016). The recognition of effectors is often dependent on R proteins that contain nucleotide binding (NB) and leucine-rich repeat (LRR) domains and are collectively known as NB-LRRs (Meyers et al., 1999). In the innate plant immune system this process is known as effector-triggered immunity (ETI; Jones and Dangl, 2006). NB-LRR genes are key to plant immunity and their presence, absence or allelic diversity is decisive for disease resistance. At least seven distinct potato NB-LRRs effective toward *P. infestans* have been cloned so far and their cognate effectors are well described (reviewed in Vleeshouwers and Oliver, 2014). Furthermore, allele mining for late blight resistance genes such as *Rpi-blb1, Rpi-blb2*, and *Rpi-blb3* from the diploid Mexican species *S. bulbocastanum* has identified functional orthologs in other species (Lokossou et al., 2009, 2010). For example, *Rpi-blb1* orthologous genes were identified in the Mexican diploid species *S. cardiophyllum*, the allopolyploid species *S. papita* and *S. polytrichon* as well as in *S. stoloniferum* amongst others (Wang et al., 2008; Lokossou et al., 2010). When seeking novel resistances in germplasm collections, it is thus imperative to exclude accessions that contain already characterized resistances as the sole means of defense against the pathogen in question.

Recent advances in genome sequencing technologies enable rapid analysis of entire crop genomes and have accelerated the identification of functional R genes. Indeed, 11 years since sequencing the model plant *Arabidopsis thaliana*, the genomes of two important *Solanaceae* crop plants, potato, and tomato, were reported (Potato Genome Sequencing Consortium (PGSC), 2011; Tomato Genome Consortium (TGC), 2012). These genomes provide a blueprint for identification of genes coding for important traits such as disease resistance. In the sequenced *Solanum tuberosum* group Phureja clone DM1-3 516 R44 (DM), 755 NB-LRR genes have been identified and their phylogenetic relationships as well as their physical locations in the 12 potato chromosomes described (Jupe et al., 2012, 2013). These studies formed the basis of a novel R gene enrichment and sequencing platform (RenSeq) that enables the improved annotation of resistance genes in sequenced genomes and facilitates rapid mapping and cloning of resistances via bulked-segregant analysis (Jupe et al., 2013).

In this study we utilized a combination of late blight infections, effector-omics, allele mining, and dRenSeq to identify and/or prioritize novel sources of resistance toward the *P. infestans* lineage 13-A2. As a proof of concept, dRenSeq was applied as a diagnostic tool to two accessions of the diploid potato species *S. okadae* and confirmed the presence of *Rpi-vnt1*.*1* in this species.

## Materials and methods

### Late blight screening of diploid CPC accessions

Isolates of *P. infestans* were established *in vivo* on leaves of the late blight susceptible cultivar Craig's Royal and passaged through several generations according to Andrivon et al. (2011). Detached leaf tests were carried out as described by Whisson et al. (2007) and seedling and whole plant tests (two replicates) as described by Stewart et al. (1983) and Bradshaw et al. (2006), respectively. Disease was scored between 5 and 8 days post infection (dpi) on a scale of resistance ranging from 1 = very susceptible to 5 = very resistant for seedling and detached leaf tests and 1 = very susceptible to 9 = very resistant; symptomless plants, for whole plants according to the Malcolmson scale (Cruickshank et al., 1982).

### Transient expression of *P. infestans* effectors in *S. okadae* accessions

*P. infestans* effectors were cloned into the binary vector pGRAB and transformed into the *A. tumefaciens* strain Agl1 with VirG and pSoup. An empty vector was used as a negative control. Infiltrations and analysis of infiltration sites were conducted as described previously (Gilroy et al., 2011).

### *Rpi-vnt1* allele mining in *S. okadae* accessions

*Rpi-vnt1-like* genes have been amplified from the *S. okadae* accessions 7129, 7625, and 7629 through PCRs with the *Rpi-vnt1* specific primers Rpi-vnt1_F_full: 5′ATGAATTATTGTGTTTACAAGACTTGG3′ and Rpi-vnt1_R_full: 5′TTATAGTACCTGTGATATTCTCAACTTTGC3′. To assess the diversity of the *Rpi-vnt1-like* sequences PCR products were cloned into the vector pGEM-T easy for Sanger sequencing, according to the manufacturer's recommendations (pGEM®-T Easy Vector System—Promega). Recombinant clones were selected following transformation of the constructs into electro competent *Escherichia coli* DH10B and DH5α cells (Invitrogen) using colony PCR with the gene specific primers mentioned above. Sequencing products were subjected to a BLASTn analysis and compared to functional *Rpi-vnt1* variants (Pel et al., 2009) using Geneious v5.6.3 (Biomatters).

### RenSeq analysis

RenSeq target enrichment and sequencing was performed according to Jupe et al. (2013, 2014) with minor modifications. The covaris sonicator M220 (Covaris), was used for the fragmentation of DNA to ~500 bp in length, with the following settings: 50 W Peak Incident Power, 20% Duty Factor, 200 cycles per burst, 60 s treatment time and 50 μL volume with 1 μg starting amount. The fragments sizes were checked using a Bioanalyser (Agilent) and no upper size selection was conducted. The samples were quantified using Qubit (Thermofisher) and the enrichment was started with 750 ng of indexed libraries. The Agilent SureSelect enrichment library utilized was designed to include all NB-LRRs identified by Jupe et al. (2013) and the sequences of the corresponding 46,220 probes can be accessed at http://solanum.hutton.ac.uk. Added to the hybridization was 1 μL of 1000 mM universal blocking primer, containing six inosines in place of the six nucleotide index sequence and a 3′ spacer C3 modification to prevent the primer from participating in any subsequent PCR amplification. The post capture amplification was performed with the Herculase II polymerase (Agilent). Sequencing was conducted on an Illumina MiSeq platform using the 2x 300 bp kit. The raw sequence reads were deposited at the European Nucleotide Archive under accession number PRJEB12834.

Paired-end Illumina MiSeq reads were first checked with FastQC (v0.10.0; Andrews, 2010) and then quality and adapter trimmed with cutadapt (v1.9; Martin, 2011) to a minimum length of 100 bp and minimum base quality of 20. The trimmed reads were then mapped to the potato DM reference genome (v4.03; Potato Genome Sequencing Consortium (PGSC), 2011; Sharma et al., 2013) or a FASTA file containing 12 cloned R genes using Bowtie2 (v2.2.1; Langmead and Salzberg, 2012) in very-sensitive end-to-end mode.

The known R genes comprise: *R1* (GenBank: AF447489.1; Ballvora et al., 2002), *R2* (GenBank: FJ536325.1; Lokossou et al., 2009), *R2-like* (GenBank: FJ536323.1; Lokossou et al., 2009), *R3a* (GenBank: AY849382.1; Huang et al., 2005), *R3b* (GenBank: JF900492.1; Li et al., 2011), *Rpi-sto1* (GenBank: EU884421.1; Vleeshouwers et al., 2008), *Rpi-pta1* (GenBank: EU884422.1; Vleeshouwers et al., 2008), *Rpi-blb1* (GenBank: AY426259.1; van der Vossen et al., 2003), *Rpi-blb2* (GenBank: DQ122125.1; van der Vossen et al., 2005, *Rpi-blb3* (GenBank: FJ536346.1; Lokossou et al., 2010), *Rpi-abpt* (GenBank: FJ536324.1; Lokossou et al., 2009), and *Rpi-vnt1.1* (GenBank: FJ423044.1; Foster et al., 2009).

For read mapping, discordant and mixed mappings were disabled and maximum insert was set to 1000 bp. Four score-min parameters were used in different mapping runs: “L,−0.03,−0.03,” “L,−0.06,−0.06,” “L,−0.3,−0.3,” and “L,−0.6,−0.6,” approximately equal to 0.5, 1, 5, and 10% mismatch rates, respectively. The resulting BAM files were sorted and indexed using SAMtools (v0.1.18; Li et al., 2009).

The percentage of mapped reads on target was calculated as the proportion of reads mapping to an annotated, targeted RenSeq region in the DM genome reference. Intersecting these RenSeq regions (plus 1000 bp up- and down-stream) against the mapped reads using BEDTools (v2.20.1; Quinlan and Hall, 2010) gave the number of on-target reads. The reads on target was then calculated as a proportion of the total number of mapped reads. Read coverage to on-target regions was estimated by dividing the number of base pairs mapped to the 704 R genes (plus 1000 bp up- and down-stream) on chromosomes 1–12 by their total length (plus 2000 bp per gene). Read coverage was also estimated for the 12 R gene reference set by dividing the total length of mapped reads by the total length of the reference set.

## Results

### Identification of diploid CPC accessions resistant to *P. infestans* genotype 13-A2

Seedlings and selected whole plants of 126 diploid CPC accessions belonging to 34 species (Supplementary Table S1) were tested with the *P. infestans* isolates 2006-3928A and/or 2009-7654A belonging to the *P. infestans* clonal lineage 13-A2. Resistance was observed within 29 of those accessions, belonging to the species *S. bulbocastanum, S. capsicibaccatum, S. microdontum, S. mochiquense, S. okadae, S. pinnatisectum, S. polyadenium, S. tarijense*, and *S. verrucosum* (Table 1). There was a strong correlation in the resistance phenotypes observed with both isolates and in the seedling vs. whole plant assays.

**Table 1:** **Seedling and whole plant late blight resistance screening results for 29 diploid accessions from the CPC**.

**Species**	**CPC accession**	**Seedling tests with 2006_3928A [1 = S to 5 = R] Mean of 2 replicates**	**Whole plant test with 2009_7654A [1 = S to 9 = R] Mean of 2 replicates**
*S. bulbocastanum*	7636	4	9
	7637	5	–
	7639		9
	7641	5	9
	7642	–	9
	7643	–	9
	7644	4	9
	7645	–	9
	7646	–	9
	7647	–	9
	7650	5	9
	7651	4	9
*S. capsicibaccatum*	7760	4.5	8.5
*S. microdontum*	3724	–	9
	3764	–	8.5
*S. mochiquense*	6021	5	–
*S. okadae*	7129	5	9
	7625	5	9
	7629	5	9
	3762^*^	5	
*S. pinnatisectum*	7521	5	–
	7659	5	–
*S. polyadenium*	7665	–	9
	7777	4.9	9
	7778	4.4	9
	7786	4.6	8
	7795	3.7	7.5
*S. tarijense*	7515	5	–
*S. verrucosum*	54	4	8

Late blight resistance was assessed on 25 4–5 week old seedlings (two replicates per test) or 9–10 weeks old selected plants from the accession (two replicates per plant) with the isolates 2006-3928A or 2009-7654A (both 13-A2), respectively. Results were recorded at 8 dpi, using a sliding scale of resistance ranging from 1 = very susceptible to 5 = very resistant for seedling tests and 1 = very susceptible to 9 = very resistant; symptomless plants, for whole plants according to the Malcolmson scale (Cruickshank et al., 1982). The resistance in accession 3762 (denoted with a ^*^) is known to be based on the presence of Rpi-vnt1.1 only.

To determine if the resistances in these species are based on novel or already characterized resistance genes, a number of complementary assays were performed. In this study we report only on accessions of *S. okadae* and tested for the presence of *Rpi-vnt1.1* amongst other characterized R genes. The resistance gene *Rpi-vnt1.1* was initially cloned from *S. venturii* and *S. okadae* as well as *S. phureja* accessions and is a homolog of the tomato mosaic virus gene *TM-2(2)* (Foster et al., 2009).

### *S. okadae* accessions respond to *Avr-vnt1* in heterologous transient expression assays

**Figure 1:**
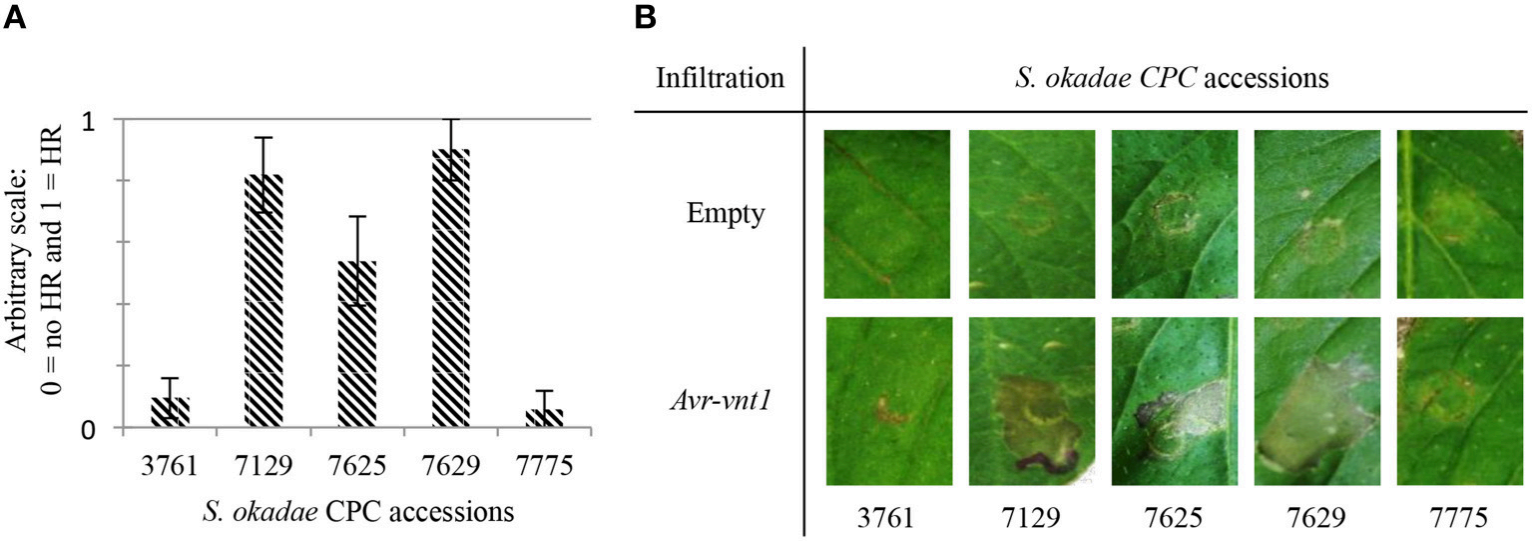
**Recognition responses following transient, *Agrobacterium tumefaciens*-based expression of *Avr-vnt1* in *S. okadae***. Accessions resistant to *P. infestans* genotype 13-A2, 7129, 7625, and 7629, yield a visible response whereas susceptible accessions 3761 and 7775 yield no specific response if compared to empty vector control. **(A)** Graph representing phenotypic response at the Avr-vnt1 infiltration sites from at least three independent replicates. Plants were scored at 5 dpi. A score of zero represent no HR and a score of one indicates that at least half the infiltrated leaf area responded with a cell death response. **(B)** Pictures of the infiltration sites of the empty vector control and Avr-vnt1 visualized under white light at 5 dpi. Transient expressions were performed by infiltration of *A. tumefaciens* strain Agl1, at an OD_600_ of 0.2.

A set of over 90 *P. infestans* RXLR effectors has been cloned into binary expressions systems to allow the heterologous expression via *Agrobacterium tumefaciens*. A subset of 82 effectors that includes known *Avr* genes (Supplementary Table S2) such as *Avr-vnt1* (Pel, 2010) was screened on accessions of *S. okadae* including susceptible plants *S. okadae* 7775 and 3761. In at least seven independent replicates with more than 14 individual infiltration sites in total, Avr-vnt1 was recognized reproducibly in *S. okadae* accessions 7129, 7625, and 7629 but not in susceptible plants 7775 or 3761 (Figure 1). *S. okadae* accession 3762 was not responsive to *Agrobacterium*-based expression of effectors and controls (data not shown).

### Allele mining and dRenSeq confirm that *S. okadae* accessions contain *Rpi-vnt1.1*

**Figure 2:**
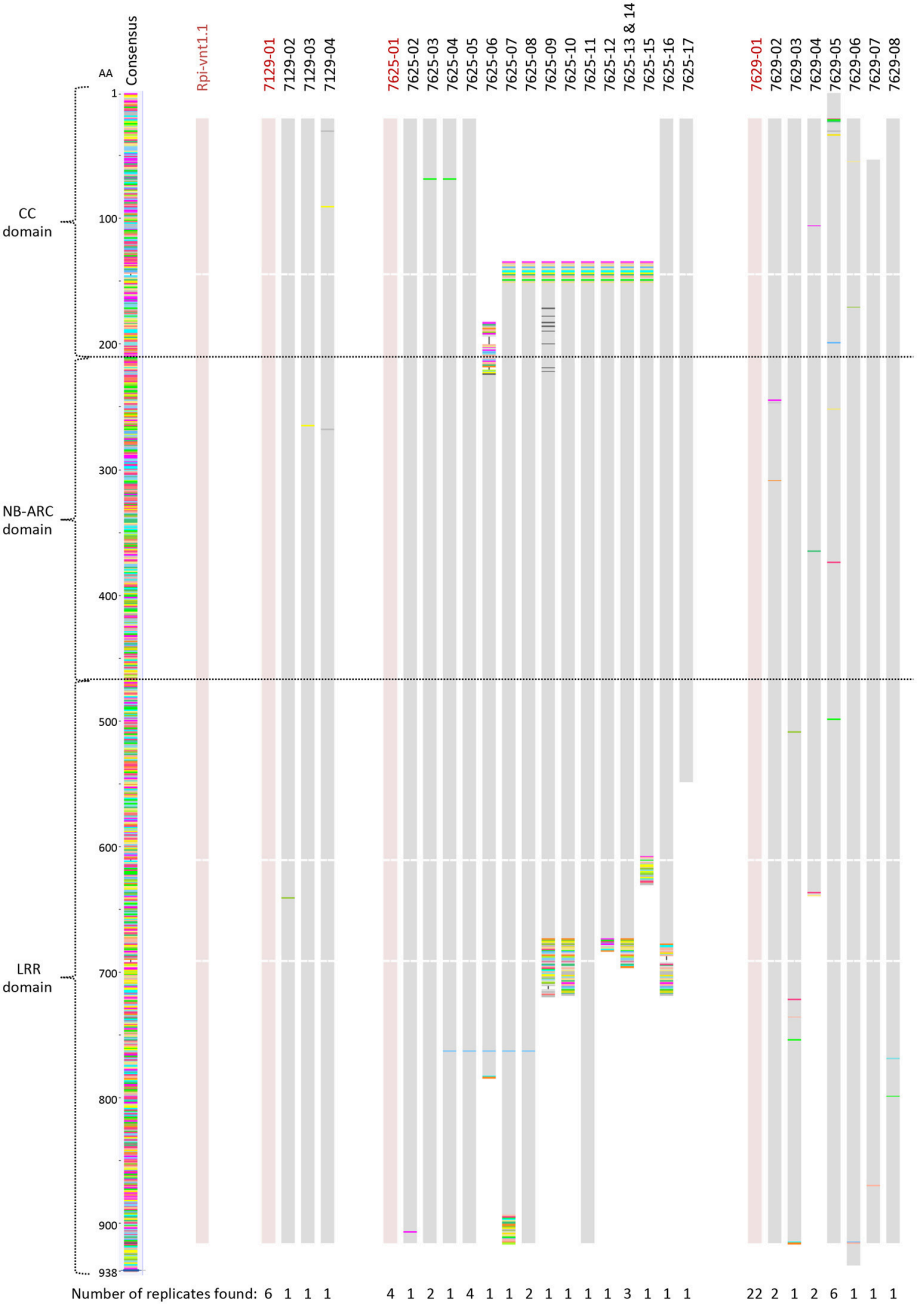
***Rpi-vnt1.1* allele mining in *S. okadae* accessions**. In total 9, 26, and 36 *Rpi-vnt1-like* genes have been amplified and sequenced from the *S. okadae* accessions 7129, 7625, and 7629, respectively. Nucleotide sequences were translated and the amino acid sequences aligned using *Rpi-vnt1.1* as a reference. Sequences identical to *Rpi-vnt1.1* are shown in red. The sequence redundancy for each clone is shown below the alignment.

*Rpi-vnt1.1* gene specific PCR primers were designed and utilized to ascertain if the *S. okadae* accessions 7129, 7625, and 7629 contain the 2676 bp long gene *Rpi-vnt1.1* (Foster et al., 2009) that is also present in *S. okadae* accession 3762 (Hein et al., unpublished). PCR products were cloned and Sanger sequenced to establish the sequences of individual clones. Alignment of PCR product sequences with *Rpi-vnt1.1* indicates that all three accessions contain a sequence identical to *Rpi-vnt1.1* alongside additional gene variations and truncated sequences (Figure 2).

**Figure 3:**
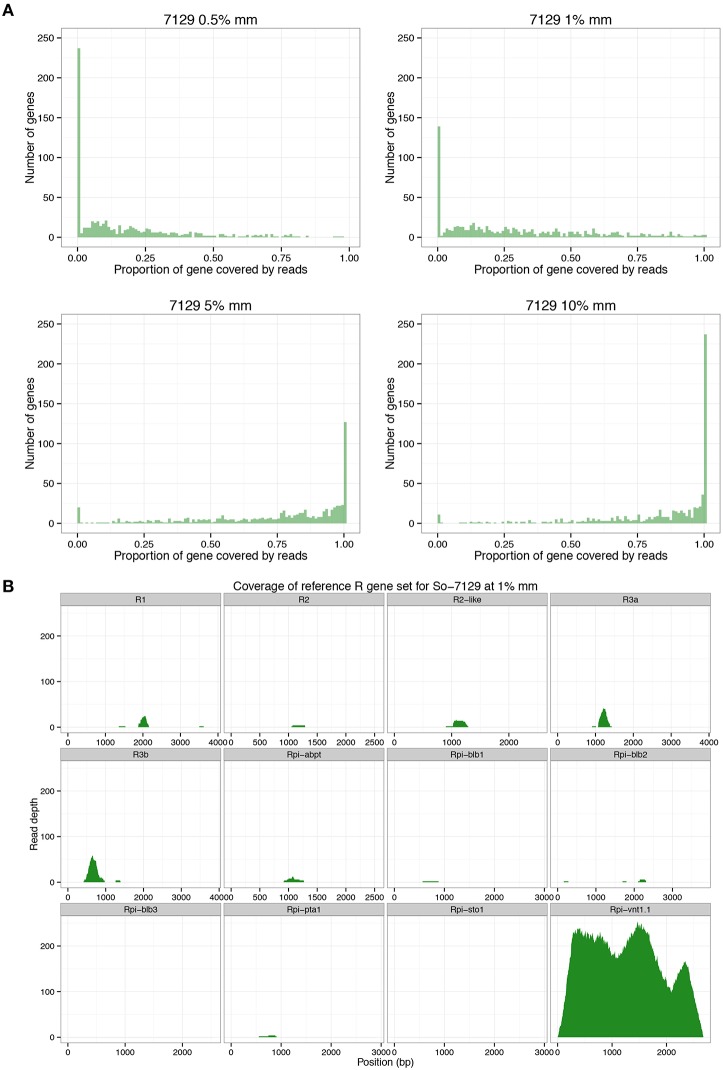
**RenSeq analysis for *S. okadae* accession 7129. (A)** The number of 704 R genes from DM with known locations on chromosomes 1–12 that are not covered (0.00), partially covered or fully covered (1.00) following RenSeq analysis in *S. okadae* accession 7129 is shown. Mismatch rates (%mm) ranging from stringent 0.5 or 1% to more relaxed 5 or 10% are displayed. **(B)** The read depth and coverage of 12 functional R genes with homologous sequences isolated from *S. okadae* accession 7129 following RenSeq analysis and mapping under stringent conditions (1% mismatch rate) are depicted.

RenSeq-based sequence analysis was conducted to corroborate the allele mining results and to establish whether RenSeq could be used as a diagnostic tool for validating the presence of functional NB-LRR genes. Genomic potato DNA samples from *S. okadae* accessions 7129 and 7625 were indexed, enriched for NB-LRR genes, and sequenced on a single lane of Illumina MiSeq. Each sample took a 12th of the MiSeq lane. Following quality control, 1,814,975 paired-end reads were obtained for *S. okadae* accession 7129 and 1,518,349 for 7625. Mapping against the sequenced potato clone DM, which has 704 NB-LRRs with known positions on chromosomes 1–12 (Jupe et al., 2013) was conducted at 0.5, 1, 5, and 10% mismatch rates. At 0.5 and 1% mismatch rates the systematic differences between *S. okadae* and *S. phureja* were apparent and a maximum of 6.49% of all reads could be mapped, of which more than 50% were on target. However, when allowing for a 5 or 10% mismatch rate, more than 46 or 70% of all reads could be mapped, respectively. Furthermore, the on-target rate increased to a maximum of 69.5% and mean coverage of NB-LRRs reached 108x (Table 2). Importantly, more of the 704 NB-LRR reference genes from DM were covered by reads from *S. okadae* accessions with conditions allowing for 5% or higher mismatch rates (Figure 3A, Supplementary Figure S1A, Table 3) indicating that the enrichment was successful.

**Table 2:** **RenSeq reads were mapped to DM genome v4.03 or a reference set of 12 R genes at various mismatch rates (% MM)**.

**CPC**	**% MM**	**Reads mapped to DM genome v4.03**					**Reads mapped to 12 functional NB-LRRs**		
		**Total**	**% Mapped**	**On target**	**% On target**	**Mean coverage (x)**	**Total**	**% Mapped**	**Mean coverage (x)**
7129	0.5	87,842	2.42	33,585	38.23	1.93	1386	0.04	9.07
	1	203,384	5.60	108,583	53.39	6.49	2034	0.06	13.36
	5	1,685,852	46.44	114,7209	68.05	72.83	50,442	1.39	328.75
	10	2,554,646	70.38	1,696,516	66.41	108.23	234404	6.46	1568.62
7625	0.5	85,054	2.80	39,880	46.89	2.22	736	0.02	4.57
	1	197,172	6.49	118,332	60.01	6.83	1214	0.04	7.26
	5	1,460,566	48.10	1,015,151	69.5	62.63	60,442	1.99	384.19
	10	2,170,588	71.48	1,472,915	67.86	91.58	256,646	8.45	1683.09

The resulting alignments were intersected (±1000 bp) against the 704 R genes from DM with known locations on chromosomes 1–12 to give the proportion of on target reads. The on target reads were then assessed for mean read coverage against the 704 genes, whilst for the 12 R gene set all the mapped reads were used to calculate the read depth.

**Table 3:** **RenSeq reads were mapped to DM genome v4.03 at 0.5, 1, 5, and 10% mismatch rates (%MM)**.

**Sample**	**% MM**	**Number of genes with % coverage**			
		**0%**	**≤5%**	**≥95%**	**100%**
7129	0.5	236	278	3	0
	1	138	167	14	3
	5	20	22	231	127
	10	11	12	340	237
7625	0.5	211	259	3	0
	1	121	156	15	3
	5	25	26	200	123
	10	15	17	318	208

The resulting alignments were cross-referenced against the 704 R genes from DM with known locations on chromosomes 1–12 to determine how many R genes were covered extensively (≥95%), completely (100%), minimally (≤ 5%), or not at all (0%).

Sequences derived from 7129 and 7625 were also mapped to a reference set of 12 characterized potato late blight NB-LRR sequences including *R1, R2, R2-like, Rpi-abpt, Rpi-blb3, R3a, R3b, Rpi-blb1, Rpi-pta1, Rpi-sto1, Rpi-blb2*, and *Rpi-vnt1.1* in a dRenSeq analysis. At 1% mismatch rate, only functional *Rpi-vnt1.1* was completely represented by dRenSeq reads (Figure 3B, Supplementary Figure S1B). Similar specific results were observed at 0.5% mismatch rate but not at 5 or 10% (Supplementary Figure S2). Indeed, at 5 and 10% mismatch rates, the mean read coverage of *Rpi-vnt1.1* was comparable to other characterized R genes (Supplementary Figure S2).

**Figure 4:**
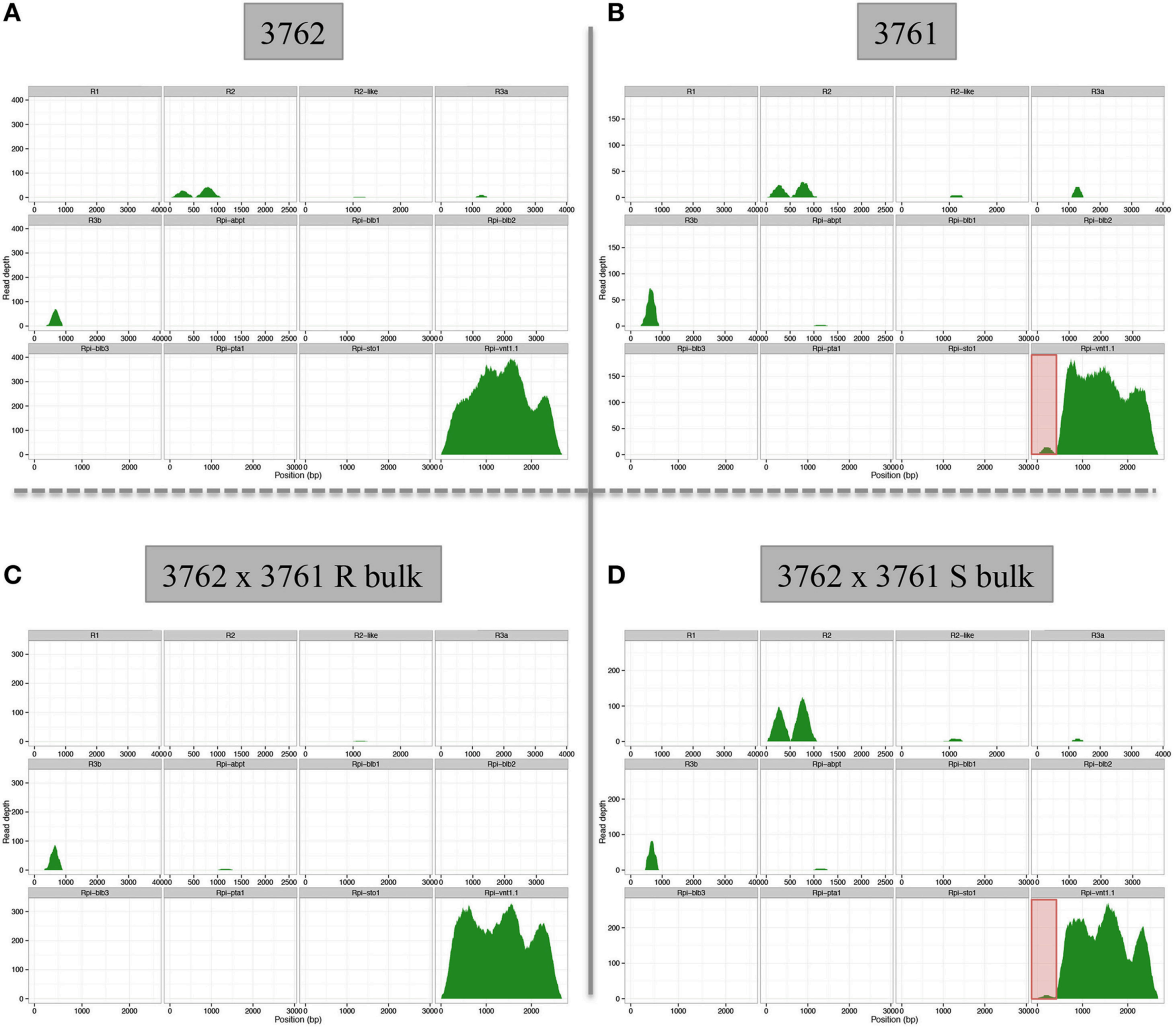
**dRenSeq analysis for resistant and susceptible *S. okadae* accession and bulked progeny**. The read depth and coverage of 12 functional R genes with homologous sequences isolated from *S. okadae* accession **(A)** 3762 carrying *Rpi_vnt1.1*, **(B)** 3761 (susceptible), **(C)** bulk of 20 resistant plants derived from a cross between 3762 and 3761, and **(D)** bulk of 20 susceptible plants derived from a cross between 3762 and 3761 following RenSeq analysis and mapping under stringent conditions (1% mismatch rate) are depicted.

Importantly, dRenSeq was also applied to resistant *S. okadae* accession 3762 (containing *Rpi-vnt1.1*) and susceptible *S. okadae* 3761 (without functional *Rpi-vnt1.1*) to validate the concept and to discern between resistant and susceptible plants from the same species. Included were also a pool of 20 resistant and 20 susceptible plants that are derived from a cross between both accessions (Figure 4, Supplementary Table S3). At a mismatch rate of either 0.5% (data not shown) or 1%, full-length *Rpi-vnt1.1* was recovered from accession 3762 and the resistant pool. However, an *Rpi-vnt1.1*-like sequence with a truncated 5′ end, compared to the functional gene, was recovered from both the susceptible accession 3761 and the susceptible pool. Indeed, the lack of sequence conservation in this region was consistently detected in both susceptible samples (Figure 4).

### *S. okadae* accessions contain additional resistance that is independent of *Rpi-vnt1.1*

Selected *S. okadae* accessions were screened with five additional *P. infestans* isolates that display broad race specificity (Supplementary Table S4). Importantly, the isolate EC1, which overcomes *Rpi-vnt1.1* resistance, was included to discern between resistances that are exclusively based on the presence of *Rpi-vnt1.1*. The potato clone *Rpi-vnt1.1*_R6, which is an F1 clone derived from the cross between *S. okadae* accessions 3762 (containing *Rpi-vnt1.1*) and 3761 (susceptible), was used as a control.

**Figure 5:**
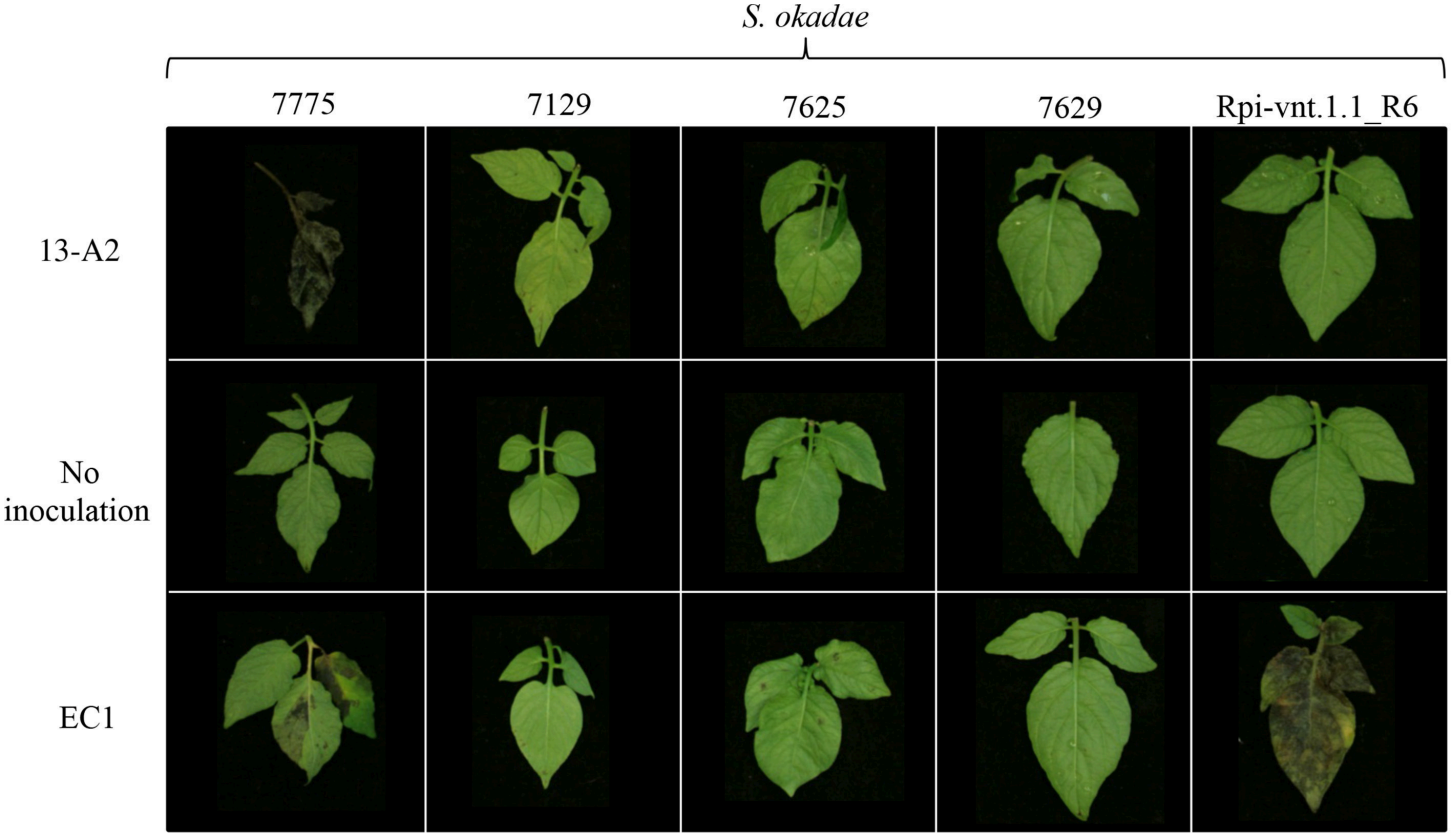
**Late blight screen of *S. okadae* accessions with EC1, a *Rpi-vnt1.1* race specific isolate of *P. infestans*, and 13-A2**. Isolates of *P. infestans* were drop-inoculated on detached leaves and symptoms assessed at 8 dpi. The *S. okadae* clone 3762-R6 has been independently characterized and only contains *Rpi-vnt1.1*, and was used as a control.

In line with previous results, the clone Rpi-vnt1.1_R6 was resistant to the 13-A2 isolate 2009-7654A and other isolates but susceptible to EC1 (Table 4). The *S. okadae* accession 7775 was susceptible to the 13-A2 isolate but partially resistant to EC1. The three *S. okadae* accessions (7129, 7625, and 7629) recognizing Avr-vnt1 (Figure 1), however, were resistant to all isolates including EC1 (Figure 5, Table 4). This provides evidence that these accessions, unlike clone Rpi-vnt1.1_R6, carry at least one additional, novel resistance gene that functions independently of *Rpi-vnt1.1*.

**Table 4:** **Late blight screen of five diploid *S. okadae* accessions from the CPC**.

**CPC accession number**	**Species or cultivars**	***P. infestans* isolates (genotype)**					
		**2009-7654A (13 A2)**	**2010-7822 (6A1)**	**2010-7814 (23A1)**	**2010-8122D (8 2 A1)**	**2010-7838A (Misc')**	**EC1 (non-characterized)**
3761	*S. okadae*	1.0	1.5	4.0	2.0	1.5	–
Rpi-vnt1.1_R6	*JHI cross*	5.0	5.0	5.0	5.0	5.0	1.0
7129	*S. okadae*	5.0	5.0	5.0	5.0	5.0	5.0
7625	*S. okadae*	5.0	5.0	5.0	5.0	5.0	4.0
7629	*S. okadae*	5.0	5.0	5.0	5.0	5.0	5.0
7775	*S. okadae*	1.0	–	–	–	–	3.0

The isolate names and genotypes are shown where known. The blight tests were performed on detached leaves using different isolates of P. infestans. Results were scored at 8 dpi, from 1 = susceptible to 5 = resistant; symptomless leaf. The scores shown are the average of at least two independent replicates. Highlighted in gray are compatible and intermediate compatible interactions.

## Discussion

Potato production is constantly threatened by late blight. The risk of infection is further exacerbated by the rapidly evolving nature of the pathogen, marked by rapid expansion of population size through asexual multiplication or increased genetic diversity through sexual reproduction. Controlling late blight by host resistance requires the continuous development of cultivars by introgression of new resistance from wild species. Breeding strategies in the 1950s largely relied on the deployment of resistances from the hexaploid species *S. demissum*, which resulted in the release of cultivars carrying one or more resistance genes. Pentland Dell, for example, a potato cultivar released in Great Britain in 1963, contained three resistance genes *R1, R2*, and *R3a* (Bradshaw and Ramsay, 2005). However, these resistances proved to be short-lived and could be overcome quite easily within 4 years by adapted new genotypes of *P. infestans* (Malcolmson, 1969). Exploration of other wild species led to the identification and cloning of resistances conferred by *Rpi-blb1* (van der Vossen et al., 2003) and *Rpi-blb2* (van der Vossen et al., 2005) from *S. bulbocastanum* and *Rpi-vnt1* from *S. venturii* (Foster et al., 2009). While these genes show a broad spectrum of resistance, there are some *P. infestans* isolates that can overcome individual R genes but not all three combined (Jones et al., 2014), showing the importance of pyramiding resistances. However, introgression of resistance genes is a long and laborious process. For example, *Rpi-blb2* has been successfully introgressed into cultivars such as Toluca and Bionica that were developed after more than 30 years of breeding and selection efforts (Haverkort et al., 2009).

In light of these observations, the need for rapid and reliable diagnostic R gene tools is apparent. Effector-omics has proven useful for breeding and the identification of orthologous R gene in wild species (Vleeshouwers et al., 2008; Vleeshouwers and Oliver, 2014; Lenman et al., 2016). However, for this system to be successful, a detailed knowledge of the recognized effector is required alongside responsive plants that yield reproducible recognition response upon transient effector expression. We have obtained reproducible Avr-vnt1 recognition responses in *S. okadae* accessions 7129, 7625, and 7629 (Figure 1) but not for 3762 that contains the cognate R gene *Rpi-vnt1.1*. The latter proved non-responsive to the transient *Agrobacterium*-based expression system.

In line with the Avr-vnt1 recognition, PCR-based allele mining and Sanger sequencing confirmed the presence of *Rpi-vnt1.1* in *S. okadae* accessions 7129, 7625, and 7629 (Figure 2). A similar approach has been utilized successfully to identify orthologous genes in wild potato species (Lokossou et al., 2009, 2010). A PCR-based screening for full-length R genes alone could, however, be prone to false-positives and/or false-negative results. Furthermore, the cloning and sequencing of PCR products, which is required to discriminate highly similar sequences (Figure 2), renders this process low to medium throughput.

This study has shown that mapping RenSeq reads with stringent mismatch rates against reference R genes, results in a quick and easy way to screen plants for the presence or absence of known R genes (Figures 3, 4, as well as Supplementary Figures S1, S2). Indeed, dRenSeq is specific enough that it could distinguish between functional *Rpi-vnt1.1* in resistant accessions and its homologs in susceptible accessions as well as bulks. As such, dRenSeq could also be used for allele mining under various stringent mapping conditions and also aid evolutionary studies. Importantly, the obtained RenSeq sequence from plants that do contain novel resistances can subsequently be used as a reference in a bulked-segregant analysis if genetic crosses can be achieved (Jupe et al., 2013). Therefore, sequence data can be used to answer different biological questions.

Interestingly, the *S. okadae* accessions 7129, 7625, and 7629 all contain functional *Rpi-vnt1.1* as demonstrated by effector recognition, allele mining and, in the case of 7129 and 7625, dRenSeq. However, they also contain a resistance that operates independent of *Rpi-vnt1.1* as demonstrated by additional late blight screening (Figure 5). The clone Rpi-vnt1.1_R6 carries *Rpi-vnt1.1* and is, as expected, resistant to blue 13 but susceptible to the isolate EC1 (Foster et al., 2009), whereas 7129, 7625, and 7629 were all resistant to both isolates (Figure 5). RenSeq-derived reads are of dual utility and the additional resistance(s) could be mapped via a bulked segregant RenSeq analysis as described in Jupe et al. (2013). In this case, the RenSeq reads that have been used for the dRenSeq analysis described here could be utilized to represent the resistant/susceptible parents. Using DM as a reference for the mapping, RenSeq reads are typically mapped at a 5% mismatch rate to allow for systematic differences between species which contrasts with dRenSeq where a 0.5 or 1% mismatch rate is used to establish the presence/absence of already known NB-LRRs.

Future efforts to identify resistances toward major pathogens in germplasm collection can quickly identify plants that contain novel resistances by taking advantage of target enrichment and sequencing technologies. For example, traditional allele mining based on PCR amplification, cloning of amplicons, and Sanger sequencing of individual clones can be omitted with dRenSeq application. Furthermore, a combination of late blight screening that includes isolates with a broad virulence spectrum followed by dRenSeq could be utilized to first prioritize plants that could subsequently be subjected to effector-omic analysis prior to a detailed genetic study. In breeding programs, dRenSeq (or similar enrichment strategies for additional genes) could be utilized to aid R gene pyramiding and/or to follow multiple important traits on a sequence-based level.

## Author contributions

PV, XC, BH, GT, AL, JL were involved in late blight screening and effector recognition. DC characterised *P. infestans* isolates. GM provided high health CPC accessions for the experiments. PV conducted allele mining and RenSeq analysis with MA. EG and PB provided effectors and were involved in analysing effector recognition. KB conducted computational analysis of RenSeq and DRenSeq. PV, GB, XC, and IH wrote the manuscript. IH directed the work.

## Funding

This work was funded by the Rural & Environment Science & Analytical Services Division of the Scottish Government, the BBSRC through the joint projects CRF/2009/SCRI/SOP 0929, BB/L008025/1, BB/ K018299/1 (IH) and the USDA NIFA grant 2011-68004-30154 (P SMVW). Additional funding was obtained through the James Hutton Institute SEEDCORN initiative.

### Conflict of interest statement

The authors declare that the research was conducted in the absence of any commercial or financial relationships that could be construed as a potential conflict of interest.
