# Supplementary material for: Utilizing “Omic” Technologies to Identify and Prioritize Novel Sources of Resistance to the Oomycete Pathogen Phytophthora infestans in Potato Germplasm Collections
Source: Front Plant Sci. 2016 May 27;7:672. doi: 10.3389/fpls.2016.00672 (PMC4882398; doi:10.3389/fpls.2016.00672)
Supplement: Supplementary file 1 [file Table1.DOCX]

Supplementary Table 1: List of diploid CPC accessions tested for late blight resistance

| Species | CPC | Species | CPC | Species | CPC |
| --- | --- | --- | --- | --- | --- |
| *S. alandiae* | 7212 | *S. ehrenbergii* | 7507 | *S. pinnatisectum* | 7661 |
|  | 7324 |  | 7510 |  | 2301 |
| *S. berthaultii* | 5701 | *S. gourlayi* | 7671 |  | 3559 |
| *S. boliviense* | 7026 |  | 7672 |  | 3863 |
|  | 7320 | *S. infundibuliforme* | 2477 |  | 7521 |
|  | 7335 | *S. kurtzianum* | 5864 |  | 7659 |
| *S. brachistotrichum* | 3822 |  | 5889 | *S. polyadenium* | 3501 |
| *S. brevicaule* | 7704 | *S. marinasense* | 6020 |  | 7665 |
|  | 7705 |  | 7616 |  | 7777 |
|  | 7709 |  | 7738 |  | 7778 |
|  | 7753 | *S. medians* | 7178 |  | 7786 |
|  | 7754 |  | 7619 |  | 7795 |
|  | 7755 | *S. megistacrolobum* | 3759 | *S. sparsipilum* | 3533 |
| *S. bulbocastanum* | 7636 | *S. michoacanum* | 3847 | *S. spegazzinii* | 3744 |
|  | 7637 |  | 7783 |  | 3745 |
|  | 7638 |  | 3740 |  | 7195 |
|  | 7641 |  | 4048 | *S. tarijense* | 7523 |
|  | 7644 |  | 4054 |  | 7207 |
|  | 7649 |  | 7160 |  | 7210 |
|  | 7650 |  | 7163 | *S. tuberosum Group Phureja* | 4188 |
|  | 7651 |  | 7174 |  | 4485 |
| *S. canasense* | 3059 |  | 7176 | *S. venturii* | 3715 |
|  | 3664 |  | 7707 |  | 7627 |
|  | 7038 |  | 7710 | *S. vernei* | 7630 |
|  | 7142 |  | 7711 |  | 7631 |
|  | 7615 |  | 7712 |  | 7789 |
|  | 7716 |  | 7714 |  | 7797 |
|  | 7720 |  | 7730 | *S. verrucosum* | 3939 |
| *S. capsicibaccatum* | 7760 | *S. mochiquense* | 6021 |  | 54 |
|  | 3554 | *S. neorossii* | 7790 |  | 5689 |
| *S. chacoense* | 7211 |  | 7628 |  | 7091 |
|  | 3057 | *S. okadae* | 3761 |  | 7213 |
|  | 3507 |  | 3762 |  | 7217 |
|  | 3886 |  | 7129 |  | 7796 |
|  | 3903 |  | 7327 | *S. violaceimarmoratum* | 7128 |
|  | 5849 |  | 7620 |  | 7611 |
|  |  |  | 7625 |  |  |
|  | 5915 |  | 7629 | *S. x doddsii* | 7040 |
|  | 5916 |  | 7775 |  | 7148 |
|  | 7234 | *S. palustre* | 2451 |  |  |
| *S. commersonii* | 5855 | *S. pampasense* | 7066 |  |  |
|  | 5858 |  | 7068 |  |  |
|  | 5861 |  | 7328 |  |  |
|  | 7058 |  | 7610 |  |  |
|  | 7520 |  |  |  |  |
