# Supplementary material for: Utilizing “Omic” Technologies to Identify and Prioritize Novel Sources of Resistance to the Oomycete Pathogen Phytophthora infestans in Potato Germplasm Collections
Source: Front Plant Sci. 2016 May 27;7:672. doi: 10.3389/fpls.2016.00672 (PMC4882398; doi:10.3389/fpls.2016.00672)
Supplement: Supplementary file 2 [file Table2.DOCX]

Supplementary Table S2: *P. infestans* effectors (PITGs) cloned into binary vector pGRAB and transformed into *A. tumefaciens* strain *Agl1.* Known *Avr* genes are denoted. Recognition responses in resistant *S. okadae* accessions 7129, 7625 and 7629 are shown. Reported are the number of responses / inoculation sites.

| **ID of PITG** | **Avr gene** | **Recognition in resistant S. okadae** | **ID of PITG** | **Avr gene** | **Recognition in resistant S. okadae** |
| --- | --- | --- | --- | --- | --- |
| PITG-04089 |  |  | PITG-13628-2 |  |  |
| PITG-04314 |  |  | PITG-13959-1 |  |  |
| PITG-06087 |  |  | PITG-14736-1 |  |  |
| PITG-06308 |  |  | PITG-14833-2 |  |  |
| PITG-06478 |  |  | PITG-(15125)15123-5 |  |  |
| PITG-14371 | *Avr3a* |  | PITG-(16240)16427-1 |  |  |
| PITG-15123 |  |  | PITG-16737-1 |  |  |
| PITG-15127 |  |  | PITG-19800-3 |  |  |
| PITG-16294 | *Avr-vnt1* | 7129 (9/11)  7625 (7/13)  7629 (9/10) | PITG-582 |  |  |
| PITG-18215 | *Avr3b* |  | PITG-2860 |  |  |
| PITG-18670 |  |  | PITG-4090 |  |  |
| PITG-20300 |  | 7625 (2/4) | PITG-4266 |  |  |
| PITG-20303 | *Avr-blb2* |  | PITG-07550-1 |  |  |
| PITG-16663 |  | 7625 (2/3) | PITG-07550-8 |  |  |
| PITG-05096 |  |  | PITG-07550-9 |  |  |
| PITG-08278 |  | 7625 (2/3) | PITG-09732-1 |  | 7625 (2/2) |
| PITG-11484 | *Avr10* |  | PITG-09732-2 |  |  |
| PITG-11507 |  | 7625 (2/3) | PITG-09732-3 |  | 7129 (3/4)  7625 (5/6)  7629 (3/3) |
| PITG-16195 |  | 7625 (2/4) | PITG-10232 |  |  |
| PITG-16726 |  |  | PITG-10540 |  | 7629 (2/3) |
| PITG-18221 |  |  | PITG-10654 |  | 7129 (3/4) |
| PITG-19617 |  |  | PITG-12731 |  |  |
| PITG-19942 |  |  | PITG-12737 |  |  |
| PITG-21778 |  |  | PITG-13093 |  |  |
| PITG-22724 |  |  | PITG-14443 |  |  |
| PITG-22798 |  |  | PITG-15110 |  |  |
| PITG-23239 |  |  | PITG-15278 |  |  |
| PITG-21388.2 | *ipiO1* |  | PITG-16705 |  | 7129 (2/2) |
| PITG-00366-1 |  |  | PITG-17063 |  |  |
| PITG-00821-2 |  |  | PITG-17309.2 |  |  |
| PITG-03192-16 |  |  | PITG-21740 |  |  |
| PITG-04097-1 |  |  | PITG-22604 |  |  |
| PITG-04339-1 |  |  | PITG-22804 |  |  |
| PITG-04388-2 |  |  | PITG-22922 |  | 7629 (2/3) |
| PITG-05750-1 |  |  | PITG-23015 |  |  |
| PITG-07689-5 |  |  | PITG-23226 |  |  |
| PITG-09585-1 |  |  | PITG-04085-1 |  |  |
| PITG-9680 |  |  | PITG-05846 |  |  |
| PITG-10673-7 |  |  | PITG-20301 |  |  |
| PITG-11383-1 |  |  | PITG-04145-2 |  | 7625 (2/3)  7629 (2/3) |
| PITG-13625-7 |  |  | vir2, 01-29 |  |  |
