# Supplementary material for: Utilizing “Omic” Technologies to Identify and Prioritize Novel Sources of Resistance to the Oomycete Pathogen Phytophthora infestans in Potato Germplasm Collections
Source: Front Plant Sci. 2016 May 27;7:672. doi: 10.3389/fpls.2016.00672 (PMC4882398; doi:10.3389/fpls.2016.00672)
Supplement: Supplementary file 3 [file Table3.DOCX]

Supplementary Table S3: RenSeq reads were mapped to DM genome v4.03 or a reference set of 12 R genes at various mismatch rates (%MM). The resulting DM alignments were intersected (+/-1000bp) against 704 R genes from DM with known locations on chromosomes 1-12 to give the proportion of on target reads. The on target reads were then assessed for mean read coverage against the 704 genes, whilst for the 12 R gene set all the mapped reads were used to calculate the read depth.

| CPC | %  MM | Reads mapped to DM genome v4.03 | | | | | Reads mapped to 12 functional NB-LRRs | | |
| --- | --- | --- | --- | --- | --- | --- | --- | --- | --- |
|  |  | Total | % Mapped | On target | %  On target | Mean coverage (x) | Total | % Mapped | Mean coverage (x) |
| 3762 | 0.5 | 174308 | 3.18 | 56341 | 32.32 | 3.62 | 2514 | 0.05 | 17.02 |
|  | 1 | 444416 | 8.10 | 249047 | 56.04 | 16.02 | 2748 | 0.05 | 18.60 |
|  | 5 | 2803274 | 51.11 | 1913685 | 68.27 | 123.05 | 72136 | 1.32 | 487.94 |
|  | 10 | 4219454 | 76.93 | 2838323 | 67.27 | 182.40 | 384018 | 7.00 | 2596.19 |
| Res. pool | 0.5 | 179834 | 2.44 | 67424 | 37.49 | 4.33 | 2302 | 0.03 | 15.57 |
|  | 1 | 529446 | 7.19 | 309808 | 58.52 | 19.93 | 2464 | 0.03 | 16.66 |
|  | 5 | 3685852 | 50.03 | 2419464 | 65.64 | 155.49 | 91688 | 1.24 | 619.93 |
|  | 10 | 5593852 | 75.92 | 3581321 | 64.02 | 229.98 | 468144 | 6.35 | 3163.43 |
| 3761 | 0.5 | 249026 | 3.92 | 67861 | 27.25 | 4.36 | 1080 | 0.02 | 7.31 |
|  | 1 | 572882 | 9.01 | 288364 | 50.34 | 18.55 | 1290 | 0.02 | 8.73 |
|  | 5 | 3280916 | 51.60 | 2131810 | 64.98 | 137.09 | 84938 | 1.34 | 574.61 |
|  | 10 | 4867230 | 76.54 | 3127877 | 64.26 | 201.02 | 437200 | 6.88 | 2956.29 |
| Sus. pool | 0.5 | 170244 | 2.62 | 57659 | 33.87 | 3.70 | 1470 | 0.02 | 9.95 |
|  | 1 | 476846 | 7.35 | 268122 | 56.23 | 17.24 | 1888 | 0.03 | 12.78 |
|  | 5 | 3189788 | 49.14 | 2065139 | 64.74 | 132.72 | 74080 | 1.14 | 500.86 |
|  | 10 | 4845936 | 74.66 | 3062376 | 63.19 | 196.67 | 395862 | 6.10 | 2675.09 |
