# Supplementary material for: Utilizing “Omic” Technologies to Identify and Prioritize Novel Sources of Resistance to the Oomycete Pathogen Phytophthora infestans in Potato Germplasm Collections
Source: Front Plant Sci. 2016 May 27;7:672. doi: 10.3389/fpls.2016.00672 (PMC4882398; doi:10.3389/fpls.2016.00672)
Supplement: Supplementary file 4 [file Table4.DOCX]

Supplementary Table 4: Late blight isolates used in the screening of *S. okadae* accessions. Shown is their name, genotype, mating type and race specificity where known.

| ***P. infestans* isolate** | **Genotype** | **Mating type** | **Race** |
| --- | --- | --- | --- |
| 2009_7654A | 13-A2_78 | A2 | R1-7, R10, R11 |
| 2010_7822B | 6-A1 | A1 | R1, R3, R4, R7, R10, R11 |
| 2010_7814A | 23-A1 | A1 | R1, R3, R4, R7 |
| 2010_8122D | 8-2-A1 | A1 | R1, R3, R4, R7, R10, R11 |
| 2010_7838A | Misc | A1 | R3, R4, R7, R11 |
| EC1 | uncharacterized | uncharacterized | Rpi-vnt1.1 |
