# Supplementary figures and images for: Utilizing “Omic” Technologies to Identify and Prioritize Novel Sources of Resistance to the Oomycete Pathogen Phytophthora infestans in Potato Germplasm Collections
Source: Front Plant Sci. 2016 May 27;7:672. doi: 10.3389/fpls.2016.00672 (PMC4882398; doi:10.3389/fpls.2016.00672)

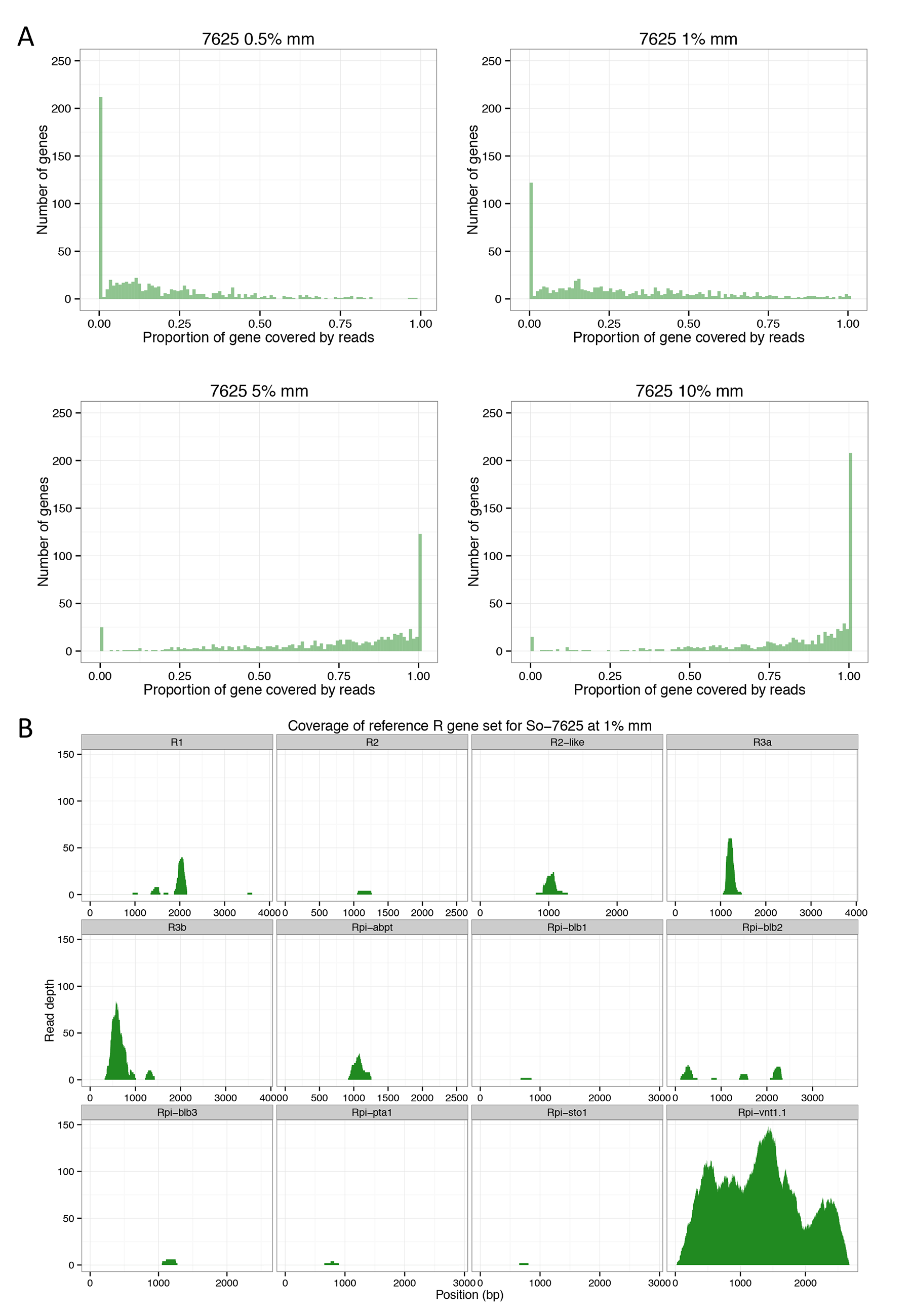

Supplement: Supplementary file 5 [file Image1.TIFF]

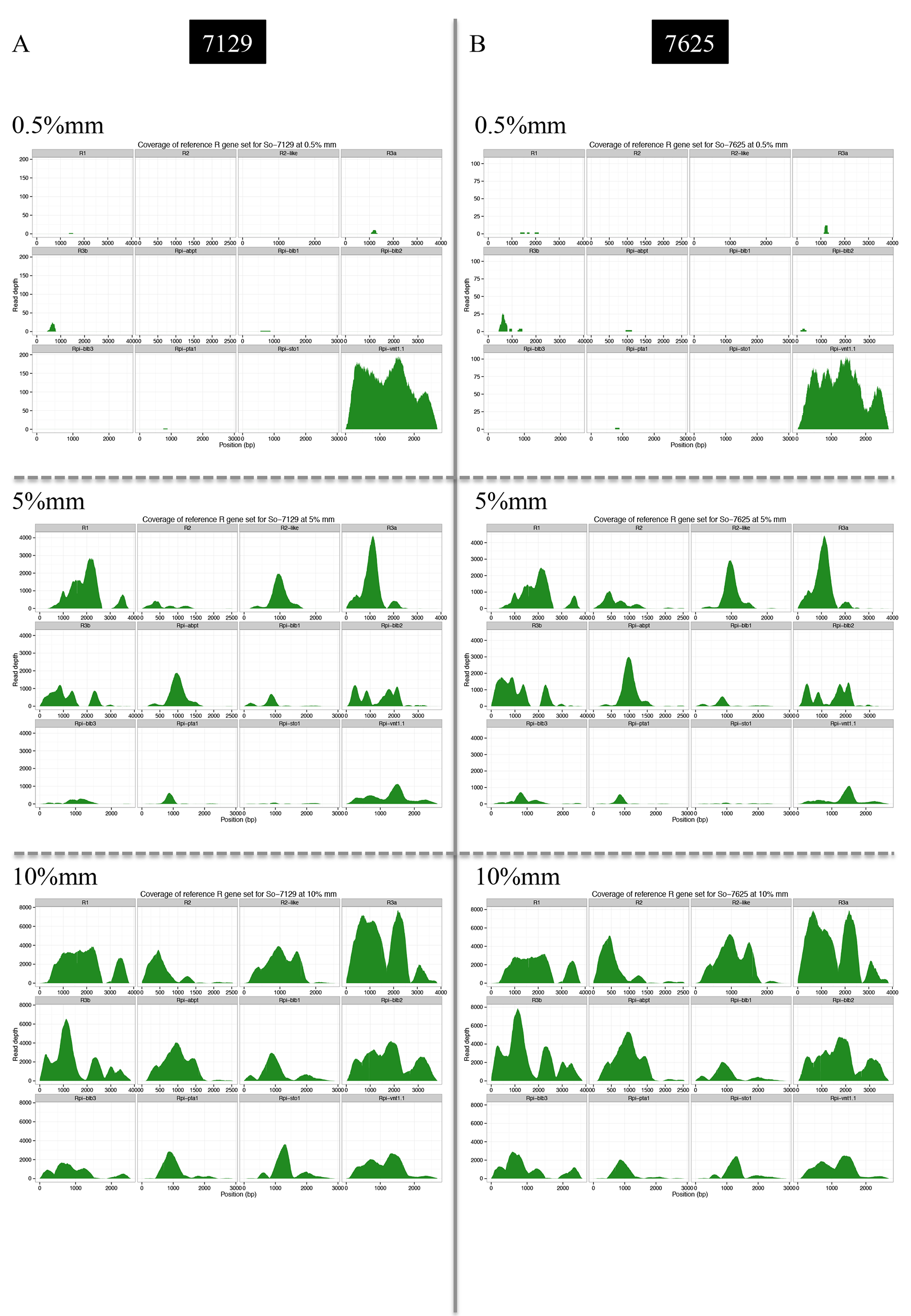

Supplement: Supplementary file 6 [file Image2.TIFF]
